# First-in-Humans Evaluation of Safety and Dosimetry of ^64^Cu-LLP2A for PET Imaging

**DOI:** 10.2967/jnumed.122.264349

**Published:** 2023-02

**Authors:** Richard Laforest, Anchal Ghai, Tyler J. Fraum, Reiko Oyama, Jennifer Frye, Helen Kaemmerer, Greg Gaehle, Tom Voller, Cedric Mpoy, Buck E. Rogers, Mark Fiala, Kooresh I. Shoghi, Samuel Achilefu, Michael Rettig, Ravi Vij, John F. DiPersio, Sally Schwarz, Monica Shokeen, Farrokh Dehdashti

**Affiliations:** 1 Alvin J. Siteman Cancer Center, Washington University School of Medicine, St. Louis, Missouri;; 2 Edward Mallinckrodt Institute of Radiology, Washington University School of Medicine, St. Louis, Missouri;; 3 Department of Radiation Oncology, Washington University School of Medicine, St. Louis, Missouri;; 4 Division of Oncology, Department of Medicine, Washington University School of Medicine, St. Louis, Missouri; and; 5 Department of Biomedical Engineering, Washington University in St. Louis, St. Louis, Missouri

**Keywords:** radiochemistry, radiopharmaceuticals, dosimetry, first-in-humans, safety, translational imaging

## Abstract

There remains an unmet need for molecularly targeted imaging agents for multiple myeloma (MM). The integrin very late antigen 4 (VLA4), is differentially expressed in malignant MM cells and in pathogenic inflammatory microenvironmental cells. [^64^Cu]Cu-CB-TE1A1P-LLP2A (^64^Cu-LLP2A) is a VLA4-targeted, high-affinity radiopharmaceutical with promising utility for managing patients diagnosed with MM. Here, we evaluated the safety and human radiation dosimetry of ^64^Cu-LLP2A for potential use in MM patients. **Methods:** A single-dose [^nat^Cu]Cu-LLP2A (Cu-LLP2A) tolerability and toxicity study was performed on CD-1 (Hsd:ICR) male and female mice. ^64^Cu-LLP2A was synthesized in accordance with good-manufacturing-practice–compliant procedures. Three MM patients and six healthy participants underwent ^64^Cu-LLP2A-PET/CT or PET/MRI at up to 3 time points to help determine tracer biodistribution, pharmacokinetics, and radiation dosimetry. Time–activity curves were plotted for each participant. Mean organ-absorbed doses and effective doses were calculated using the OLINDA software. Tracer bioactivity was evaluated via cell-binding assays, and metabolites from human blood samples were analyzed with analytic radio–high-performance liquid chromatography. When feasible, VLA4 expression was evaluated in the biopsy tissues using 14-color flow cytometry. **Results:** A 150-fold mass excess of the desired imaging dose was tolerated well in male and female CD-1 mice (no observed adverse effect level). Time–activity curves from human imaging data showed rapid tracer clearance from blood via the kidneys and bladder. The effective dose of ^64^Cu-LLP2A in humans was 0.036 ± 0.006 mSv/MBq, and the spleen had the highest organ uptake, 0.142 ± 0.034 mSv/MBq. Among all tissues, the red marrow demonstrated the highest residence time. Image quality analysis supports an early imaging time (4–5 h after injection of the radiotracer) as optimal. Cell studies showed statistically significant blocking for the tracer produced for all human studies (82.42% ± 13.47%). Blood metabolism studies confirmed a stable product peak (>90%) up to 1 h after injection of the radiopharmaceutical. No clinical or laboratory adverse events related to ^64^Cu-LLP2A were observed in the human participants. **Conclusion:** ^64^Cu-LLP2A exhibited a favorable dosimetry and safety profile for use in humans.

## Visual Abstract

**Figure:**
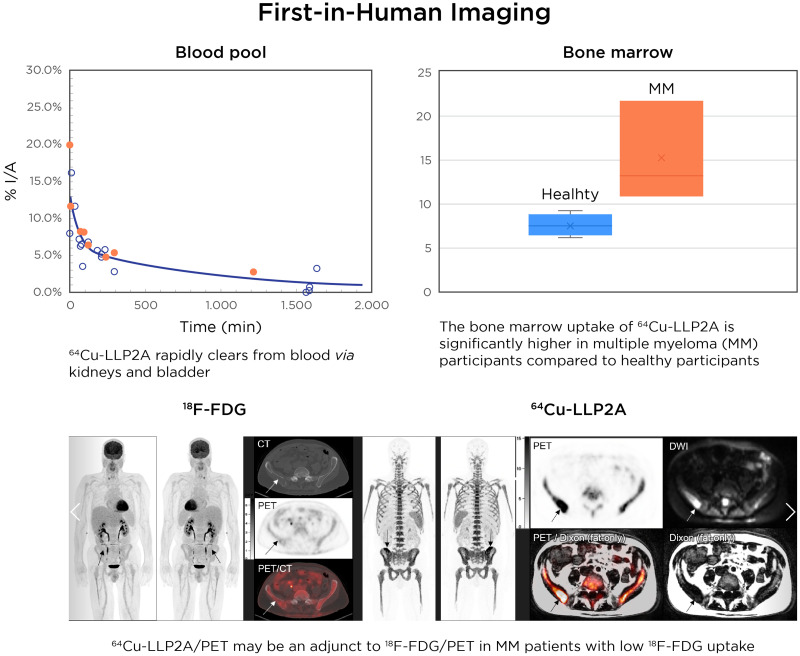

Recent advances in molecularly targeted radiopharmaceuticals have been nothing short of transformative. In oncology, PET imaging using molecularly targeted radiolabeled molecules is a vital approach toward managing patients effectively and improving outcomes. Widespread development of diverse disease-specific small molecules, peptides, and antibodies as imaging vectors has thrust discovery of new oncogenic molecular mechanisms and biomarkers.

PET imaging performed with the metabolic radiopharmaceutical, ^18^F-FDG, has been the leading nuclear medicine tracer for oncologic studies, as demonstrated by its wide availability and frequent use (1). Of note, as compared with solid tumors, in hematologic malignancies ^18^F-FDG PET/CT remains the mainstay for imaging of extramedullary infiltration, relapse, and assessment of inflammatory activity in leukemia as well as in ^18^F-FDG–avid lymphoma (2). There is increasing evidence of superior accuracy when nuclear imaging is synergized with liquid biopsies (3). Multiple myeloma (MM) is the second most common hematologic cancer, can cause debilitating end-organ symptoms, and remains largely incurable. It is a disease of malignant plasma cells that originate in the bone marrow (BM). Myeloma is commonly preceded by either or both precursor states: monoclonal gammopathy of undetermined significance and smoldering MM. Although the precursor states are not symptomatic; they are not benign either, and they present with a variable progression rate to overt myeloma. The unstable genome (4); inter-, intra-, and spatial tumoral heterogeneity (5); and age, race, and immunosuppressive BM microenvironment all contribute to the complexity and nonuniformity of myeloma pathogenesis. Consequently, the therapy options for MM encompass a combination of corticosteroids, immunomodulatory agents, proteasome inhibitors, immune- and cell-based therapies, and BM transplantation (6).

One of the overt features of MM is the presentation of diffuse infiltration and punctate focal lesions in the BM (7). Molecular PET and functional MRI are highly informative in the management of patients with MM, from initial diagnosis to therapy and longitudinal tumor monitoring (8), especially in the context of bone and BM involvement (9). Anatomic and functional imaging play a forefront role in the detection of minimal residual disease and relapse and in therapy response as well (10). ^18^F-FDG PET/CT works adequately in patients in whom ^18^F-FDG–avid myeloma lesions manifest (11). Despite its frequent use in the clinic, a known limitation of ^18^F-FDG PET in MM is the inconsistent expression of glucose transporter 1 and hexokinase 2 enzyme in myeloma cells (12). ^18^F-FDG uptake can additionally change during the course of disease progression and after therapy (12*,*^13^). Collectively, the tumor microenvironment in MM can significantly affect ^18^F-FDG signal specificity in the BM, leading to either an overestimation or an underestimation of disease burden. Therefore, development of new molecularly targeted tracers for imaging and therapy of MM that can supplement these limitations is the logical next step. It is encouraging to witness an exciting array of new tracers (14) in MM targeted toward metabolism and altered proteins such as CD38, CXCR4, and BCMA (15–18).

Myeloma cells thrive on the pathogenic interactions with the cellular and noncellular components of the BM. One of the molecules that contributes significantly to the vicious cycle of the MM–BM interaction is the integrin very late antigen 4 (VLA4). VLA4 is overexpressed on MM cells relative to other cells and is an established marker of cell adhesion–mediated drug resistance. We recently described the transcriptomic and biologic effects of VLA4 modulation in myeloma cells (19). Encouragingly, there is a well-characterized preclinical PET probe, [^64^Cu]Cu-CB-TE1A1P-LLP2A (^64^Cu-LLP2A), specific to the activated conformation of VLA4 (20*,*^21^). We have previously demonstrated the utility of ^64^Cu-LLP2A-PET in diverse human and mouse models of MM (19*,*^22^–^24^).

After rigorous characterization; in vitro and in vivo evaluations; and rodent dosimetry, toxicity, and safety studies, we initiated and completed the first-in-humans evaluation of ^64^Cu-LLP2A with the primary goal of evaluating the safety and dosimetry of this tracer. The secondary goal was to determine the optimal imaging time point in humans. Furthermore, this foundational study informs optimization of the next generation of VLA4-targeted radiotracers for achieving a precise signal-to-background ratio in the BM.

## MATERIALS AND METHODS

LLP2A-CB-TE1A1P (LLP2A) peptide was purchased from Auspep Pyt (Tullamarine). All other chemicals were purchased from Sigma Aldrich unless otherwise noted. ^64^Cu was purchased from Washington University School of Medicine and was produced on a TR-19 biomedical cyclotron (Nuclear System Co.) at the Washington University School of Medicine. The institutional review board approved the study, and all participants gave written informed consent. All data in the tables and all radiopharmaceutical doses mentioned are correct.

### Good-Manufacturing-Practice–Compliant Synthesis of ^64^Cu-LLP2A for Human PET Imaging

^64^Cu-LLP2A was synthesized according to the good-manufacturing-practice–compliant procedure in a chemistry hot cell. Details are provided in the supplemental materials and Supplemental Figure 1 (supplemental materials are available at http://jnm.snmjournals.org).

### Prerelease and Postrelease Quality Control Specifications

^64^Cu-LLP2A was released for clinical use after prerelease quality control specifications were met. Pre- and postrelease conditions are described in the supplemental materials and Supplemental Figure 2.

### Preclinical Studies

#### Cell Uptake Assay

A whole-cell uptake assay was performed as previously described to calculate the percentage specific uptake of ^64^Cu-LLP2A in the murine MM cell line, 5TGM1 (24). Additional details are provided in the supplemental methods.

#### Toxicity Studies on Mice

The toxicity of [^nat^Cu]Cu-LLP2A (Cu-LLP2A) was evaluated in male and female CD-1 IGS mice. Details are in the supplemental methods.

### Clinical Studies

#### Patient Population

We studied 6 healthy participants and 3 participants with a confirmed diagnosis of MM (Table 1). This study (NCT03804424) was approved by the Institutional Review Board and the Radioactive Drug Research Committee at Washington University School of Medicine and was conducted under investigational new drug application 136782 submitted to the U.S. Food and Drug Administration. All patients gave written informed consent before participation. The inclusion criteria for patients with MM included an age of 18 y or older and clinically or pathologically defined MM in accordance with the criteria of the International Myeloma Working Group. All types of active myeloma were eligible, including both newly diagnosed and previously treated. Healthy volunteers were eligible if the study principle investigator assessed them as being healthy, if they were at least 18 y old, and if they had no known hematologic disorder (e.g., anemia or leukemia).

**TABLE 1.** Participant Characteristics

Subject no.	Sex	Age (y)	Weight (kg)	Dose injected (MBq)	Scanner	Participant status*	Preinjection mDRD glomerular filtration rate^†^ (mL/min/1.73 m^2^)
MMDN01	F	44	68	257.5	mMR	Healthy	69.8
MMDN02	M	30	90.7	357	mMR	Healthy	82
MMDN03	F	25	61.2	347	mMR; Bio40 PET/CT	Healthy	76.3
MMDN04	F	25	63	370	mMR	Healthy	90
MMDN05	M	25	77.1	433	mMR	Healthy	112.9
MMDN06	F	27	66.7	361.5	mMR	Healthy	91.3
MMDM01	F	83	54	377	mMR	MM: untreated; plasma cell burden, 24%; ISS, I; R-ISS, II; subtype, IgA κ; cytogenetics, t (14*,*^16^)	70.5
MMDM02	M	63	77.1	366	mMR	MM: untreated; plasma cell burden, 22%; ISS, UNK; R-ISS, UNK; subtype, IgG κ; no high-risk abnormalities	52.5
MMDM03^‡^	M	68	136.1	301.2	Vision PET/CT	MM: relapsed; plasma cell burden, 20%; ISS, 3; R-ISS, 3; subtype, IgG λ; cytogenetics, t (4*,*^14^)	40.3

* MM status includes BM plasma cell infiltration, clinical parameters, disease stage, and subtype.

^‡^ Prior treatments for MMDM03 include (1) bortezomib, lenalidomide, and dexamethasone; melphalan autologous stem cell transplantation; and lenalidomide maintenance; (2) lenalidomide and dexamethasone; daratumumab, bortezomib, and dexamethasone; and pomalidomide and dexamethasone; (3) daratumumab, carfilzomib, and dexamethasone; and (4) elotuzumab, pomalidomide, and dexamethasone.

^†^ Glomerular filtration rate reference: https://www.mdcalc.com/mdrd-gfr-equation.

mDRD = modification of diet in renal disease equation; ISS = MM international staging system; R-ISS = revised MM international staging system; UNK = unknown.

For safety evaluation, the vital signs of all participants were measured (blood pressure, heart and respiratory rate, and temperature) and they underwent clinical laboratory testing (standard hematologic and comprehensive metabolic panels that included measurement of hemoglobin, white blood cells, neutrophils, lymphocytes, platelets, creatinine, blood urea nitrogen, calcium, sodium, potassium, carbon dioxide, alanine transaminase, aspartate aminotransferase, alkaline phosphatase, total bilirubin, and albumin), urinalysis, and electrocardiography before ^64^Cu-LLP2A administration and at 60 min after injection or before discharge. All participants were additionally monitored for adverse reactions (e.g., dyspnea, chest tightness, fever, and rigors) during administration of ^64^Cu-LLP2A.

#### PET Imaging Procedures

PET imaging was performed with a Siemens mMR, Biograph 40HD PET/CT, or Biograph Vision scanner. The scanners were independently calibrated against a National Institute of Standards and Technology–traceable ^68^Ge source and then were cross calibrated to the dose calibrator using an F08 water cylinder. Participants entering the study were asked to undergo ^64^Cu-LLP2A-PET at up to 3 separate time points to calculate human dosimetry. Two of the MM participants underwent ^64^Cu-LLP2A-PET/MRI, and one of the MM participant underwent ^64^Cu-LLP2A-PET/CT. All healthy participants underwent ^64^Cu-LLP2A-PET/MRI, and 1 healthy participant underwent CT (^64^Cu-LLP2A-PET/CT) at the third imaging time point. All participants were injected with ^64^Cu-LLP2A at median dose of 352.24 MBq (range, 247–433 MBq). Three participants underwent a single-station 60-min dynamic study immediately after administration of ^64^Cu-LLP2A over the known site of the tumor (1 MM participant) or over the lower lumbar spine and pelvis (2 healthy participants). Dynamic imaging was followed by static body imaging (typically mid brain to lower thighs) at 2 time points. The remaining 6 participants did not undergo dynamic imaging but rather underwent static body imaging at 3 time points between 0 and 26 h after injection. ^64^Cu-LLP2A-PET/MR or PET/CT images were evaluated to determine the imaging time after administration of ^64^Cu-LLP2A that yields the best-quality images and the best tumor-to-nontumor ratio for visual and quantitative analysis of the images. ^64^Cu-LLP2A-PET images were correlated with all available imaging studies to assess lesion uptake of ^64^Cu-LLP2A in known lesions seen on the radiologic studies.

PET/MRI in all participants consisted of a 2-point DIXON MRI for attenuation correction and body emission scans (2–5 min per bed position). In all participants who underwent simultaneous PET/MRI, additional sequences were performed: T1-weighted turbo-spin echo (TSE), T2-weighted fat suppression post-contrast imaging, diffusion-weighted imaging (DWI)/apparent diffusion coefficient (ADC) dynamic imaging, and contrast-enhanced (DCE) imaging.

PET/CT consisted of a spiral CT scan for attenuation correction (120 kVp, 50 effective mAs at a 4-mm slice thickness) from the top of the skull through the upper thighs with the subject supine. Emission images beginning at the skull and proceeding through the lower thighs were obtained (at a rate of 1–10 min per bed position, depending on the time after injection) over 6–7 bed positions with a total imaging duration of no more than 1 h. Images were reconstructed with 3-dimensional ordered-subsets expectation maximization with 3 iterations, 21 subsets, and a postreconstruction gaussian filter of 4 mm.

#### Image Analysis

Two nuclear medicine physicians and 1 physicist reviewed the images. Similar findings were found by all 3 reviewers, independently. The quantitative analysis was performed primarily by 1 individual, with cross-validation and contributions from the other 2 experts. PET images of the healthy participants were evaluated to assess the biodistribution of ^64^Cu-LLP2A. The images of the patients diagnosed with MM were evaluated qualitatively in comparison with the healthy participants with the following grading scale: no uptake (tumor ≤ background), minimal uptake (tumor = background), moderate uptake (tumor > background), and intense uptake (tumor ≫ background). The images were evaluated semiquantitatively by measurement of the tumor SUV_max_. A region of interest was drawn around the entire lesion, with knowledge of the tumor location. In patients with no focal lesions and positive BM biopsy results for the iliac bone for MM, we determined SUV_max_, SUV_mean_, and the iliac bone (or tumor)–to–spleen (and liver) ratio. The SUV for BM uptake was measured as the average of lumbar vertebrae 3–5 in most participants (supplemental materials). For radiation dosimetry estimation, volumes of interest (VOIs) were traced on the organs on the PET images with visible uptake. The liver, spleen, and kidney average organ activity concentration was measured by drawing a VOI that encompassed most of the organs as visible on the PET images at each imaging time point. The blood-pool activity was measured from a VOI traced with the left ventricle of the heart. Red marrow activity was measured from the tracer’s VOIs on the marrow uptake seen on lumber vertebrae 2–4. The total organ activity was then scaled by the standard male or female organ masses as defined by the International Commission on Radiological Protection (25). Total urinary bladder content was measured from a VOI encompassing the whole bladder as seen on the PET images.

#### Organ Time-Integrated Activity and Radiation Dose

**FIGURE 1.**
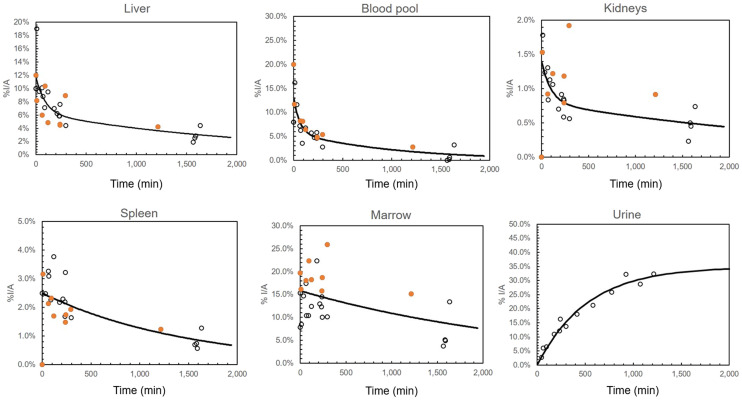
Aggregated time–activity curves. Orange circles indicate MM participants; black circles indicate healthy participants. %I/A = percentage injected activity.

Organ time-integrated activity was calculated on a per-patient basis by analytic integration of mono- or dual-exponent fits for the liver, spleen, kidneys, marrow, and blood-pool time–activity curves. The heart content time-integrated activity was computed from the blood time-integrated activity and the total blood volume and heart chamber volume for the adult male or female as defined in International Commission on Radiological Protection publication 106 (25). The cumulative urine data (from both imaging and urine collection) were plotted as a function of time and were fitted for each patient with an uptake function of the form (*A* = *A*_0_(1 − exp(−*A*_1_ *t*)), where *A*_0_ is the filling fraction and *A*_1_ is related to the filling half-life by the relation ln(2)/*A*_1_. The filling fraction and filling half-life were then entered in the MIRD bladder voiding model along with a voiding interval of 2 h to yield the bladder content time-integrated activity (26). Aggregated time–activity curves are presented in Figure 1. The male and female radiation doses, the average organ radiation doses per sex, and the effective dose were generated (Table 2). The organ residence times calculated from the human dosimetry data are presented in Table 3. Additional details are included in the supplemental materials.

**TABLE 2.** Organ Radiation Dose (*n* = 9)

										Sex		
Organ	MMDM01 (F)	MMDM02 (M)	MMDM032 (M)	MMDN012 (M)	MMDN022 (M)	MMDN03 (F)	MMDN04 (F)	MMDN052 (M)	MMDN06 (F)	Average	M	F
Adrenals	0.029	0.024	0.022	0.027	0.026	0.023	0.018	0.017	0.011	0.022 ± 0.006	0.023 ± 0.004	0.020 ± 0.008
Brain	0.020	0.017	0.015	0.020	0.020	0.015	0.012	0.013	0.004	0.015 ± 0.005	0.017 ± 0.003	0.013 ± 0.006
Breasts	0.018		0.000			0.014	0.012		0.004	0.010 ± 0.008		0.012 ± 0.006
Gallbladder wall	0.026	0.022	0.021	0.028	0.026	0.021	0.017	0.017	0.009	0.021 ± 0.006	0.023 ± 0.004	0.018 ± 0.007
Lower large intestine wall	0.026	0.022	0.020	0.025	0.024	0.021	0.017	0.017	0.009	0.020 ± 0.005	0.022 ± 0.003	0.018 ± 0.007
Small intestine wall	0.025	0.022	0.020	0.026	0.025	0.019	0.015	0.016	0.008	0.020 ± 0.006	0.022 ± 0.004	0.017 ± 0.007
Stomach wall	0.023	0.021	0.019	0.025	0.024	0.018	0.015	0.015	0.006	0.018 ± 0.006	0.021 ± 0.004	0.016 ± 0.007
Upper large intestine wall	0.025	0.021	0.019	0.025	0.024	0.020	0.016	0.016	0.007	0.019 ± 0.006	0.021 ± 0.004	0.017 ± 0.007
Heart wall	0.029	0.026	0.023	0.028	0.026	0.024	0.018	0.016	0.009	0.022 ± 0.007	0.024 ± 0.005	0.020 ± 0.009
Kidneys	0.042	0.063	0.052	0.043	0.084	0.048	0.042	0.028	0.044	0.050 ± 0.016	0.054 ± 0.021	0.044 ± 0.003
Liver	0.073	0.035	0.044	0.064	0.039	0.068	0.054	0.037	0.055	0.052 ± 0.014	0.044 ± 0.012	0.063 ± 0.009
Lungs	0.022	0.019	0.017	0.023	0.022	0.018	0.014	0.014	0.006	0.017 ± 0.005	0.019 ± 0.003	0.015 ± 0.007
Muscle	0.021	0.018	0.017	0.022	0.021	0.016	0.013	0.014	0.005	0.016 ± 0.005	0.018 ± 0.003	0.014 ± 0.006
Ovaries	0.026		0.000			0.021	0.017		0.009	0.015 ± 0.010		0.018 ± 0.007
Pancreas	0.027	0.023	0.022	0.029	0.027	0.022	0.017	0.018	0.009	0.021 ± 0.006	0.024 ± 0.004	0.019 ± 0.007
Red marrow	0.165	0.123	0.113	0.088	0.081	0.121	0.070	0.064	0.112	0.104 ± 0.032	0.094 ± 0.024	0.117 ± 0.039
Skeleton	0.158	0.093	0.085	0.080	0.076	0.117	0.074	0.055	0.092	0.092 ± 0.030	0.078 ± 0.014	0.110 ± 0.037
Skin	0.017	0.016	0.014	0.019	0.018	0.013	0.011	0.012	0.004	0.014 ± 0.005	0.016 ± 0.003	0.011 ± 0.006
Spleen	0.132	0.123	0.124	0.220	0.138	0.170	0.111	0.116	0.141	0.142 ± 0.034	0.144 ± 0.043	0.138 ± 0.025
Testes		0.017	0.016	0.021	0.020			0.013		0.018 ± 0.003	0.018 ± 0.003	
Thymus	0.021	0.019	0.017	0.022	0.021	0.016	0.013	0.014	0.004	0.016 ± 0.005	0.018 ± 0.003	0.014 ± 0.007
Thyroid	0.020	0.018	0.016	0.022	0.021	0.015	0.012	0.013	0.004	0.016 ± 0.006	0.018 ± 0.003	0.013 ± 0.007
Urinary bladder wall	0.070	0.058	0.064	0.062	0.051	0.129	0.127	0.098	0.186	0.094 ± 0.045	0.067 ± 0.018	0.128 ± 0.047
Uterus	0.025	0.000	0.000	0.000	0.000	0.021	0.017	0.000	0.010	0.008 ± 0.010		0.018 ± 0.006
Lens of eyes	0.029	0.023	0.021	0.026	0.024	0.023	0.018	0.017	0.012	0.021 ± 0.005	0.022 ± 0.004	0.021 ± 0.007
Total body	0.029	0.023	0.021	0.026	0.025	0.023	0.018	0.016	0.012	0.021 ± 0.005	0.022 ± 0.004	0.020 ± 0.007
Effective dose equivalent (Sv/MBq)	0.058	0.047	0.044	0.051	0.045	0.054	0.040	0.035	0.046	0.047 ± 0.007	0.044 ± 0.006	0.049 ± 0.008
Effective dose (Sv/MBq)	0.046	0.038	0.036	0.040	0.035	0.043	0.030	0.029	0.032	0.036 ± 0.006	0.035 ± 0.004	0.038 ± 0.008

MM participants are MMDM01, MMDM02, and MMDM03; healthy participants are MMDN01, MMDN02, MMDN03, MMDN04, MMDN05, and MMDN06. Data are Sv/MBq.

**TABLE 3.** Residence Times Calculated from Human Dosimetry Data (*n* = 9)

Organ	MMDM01 (F)	MMDM02 (M)	MMDM03 (M)	MMDN01 (M)	MMDN02 (M)	MMDN03 (F)	MMDN04 (F)	MMDN05 (M)	MMDN06 (F)
Liver	1.05	0.62	0.82	1.23	0.71	1.00	0.80	0.71	0.83
Kidneys	0.10	0.20	0.16	0.12	0.28	0.13	0.12	0.08	0.13
Bladder content	3.21	3.29	4.34	1.08	2.10	6.72	9.25	7.46	12.48
Spleen	0.23	0.19	0.27	0.48	0.18	0.31	0.20	0.25	0.26
Red marrow	4.75	3.11	2.89	2.02	1.83	2.93	1.89	1.54	1.56
Heart content	0.072	0.053	0.061	0.055	0.041	0.067	0.042	0.015	0.035
Total	9.41	7.47	8.54	4.99	5.15	11.16	12.30	10.05	15.28
MIRD 2-h void	0.19	0.20	0.24	0.10	0.15	0.43	0.48	0.43	0.68
Excreted	3.02	3.09	4.10	0.99	1.95	6.29	8.77	7.03	11.79
Remainder	8.91	10.85	9.78	13.34	13.18	7.17	6.02	8.27	3.05

MM participants are MMDM01, MMDM02, and MMDM03; healthy participants are MMDN01, MMDN02, MMDN03, MMDN04, MMDN05, and MMDN06. Data are hours.

#### Blood Metabolism Study

To determine the stability of ^64^Cu-LLP2A and to measure the metabolites of ^64^Cu-LLP2A in human samples, whole blood samples were collected and analyzed by analytic radio–high-performance liquid chromatography. Additional details are in the supplemental methods.

#### Flow Cytometry Study

Two of the 3 participants with MM agreed to provide blood and BM samples for an institutional banking study of plasma cell dyscrasias. For this study, a sample from one of the MM patients was analyzed. Details are provided in the supplemental methods.

### Statistical Analysis

Student *t* tests were used to evaluate differences in tracer uptake between patients with MM and healthy participants. Demographic and clinical characteristics were summarized by descriptive statistics. A *P* value of less than  0.05 was deemed statistically significant.

## RESULTS

### Cell-Binding Assays

The whole cell uptake of ^64^Cu-LLP2A at 37°C in VLA4-expressing 5TGM1 cells was determined as part of the postrelease quality control after each radiolabeling study. Cell uptake of ^64^Cu-LLP2A was significantly reduced in the presence of the blocking agent (unlabeled LLP2A) (*n* = 9; percentage blocking, 82.42 ± 13.47) (Supplemental Fig. 3).

### Animal Toxicity Studies

A single intravenous injection of 0.0103 mg of Cu-LLP2A per mouse was well tolerated in male and female CD-1 IGS mice and was considered a level with no observed adverse effects (Supplemental Fig. 4). Additional details are provided in the supplemental results.

### Human Imaging

Six healthy volunteers and 3 participants with a confirmed MM diagnosis (median age, 30 y; range, 25–83 y) participated in the study. On the basis of no-observed-adverse-effect-level data, the maximum administered amount of VLA4-targeted radiotracer, ^64^Cu-LLP2A, was calculated to be 15 μg, and the mean radioactivity administered per patient was 352.24 MBq (9.5 μg), with a maximum specific activity of 572.7 MBq/nmol. The radiochemical purity of more than 90% was confirmed by radio–high-performance liquid chromatography for all participant imaging sessions.

**FIGURE 2.**
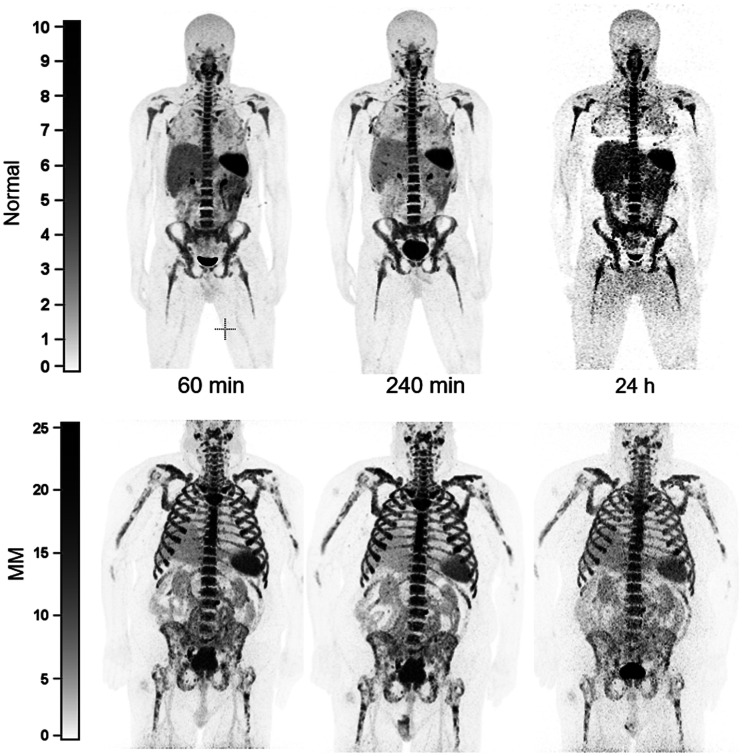
Anterior maximum-intensity-projection ^64^Cu-LLP2A-PET images of healthy volunteer (MMDN05) and subject with MM (MMDM03) at similar time points after tracer injection. Best-quality images were obtained 1–5 h after injection of radiotracer. Later time points (∼24 h) exhibited relatively lower count and high image noise. Unit of measurement for intensity bars = SUV.

**FIGURE 3.**
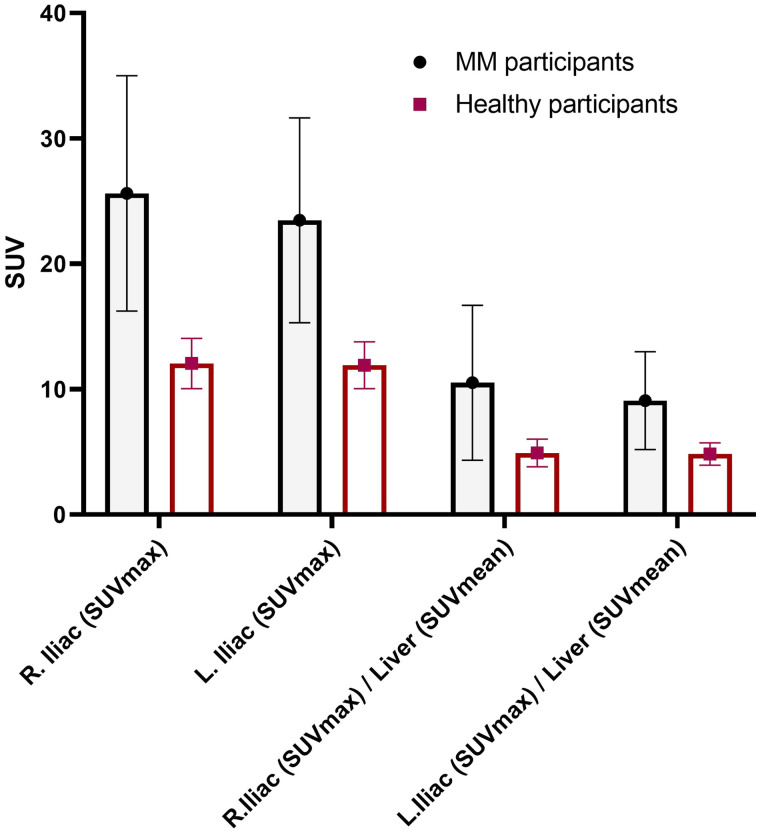
Comparison of ^64^Cu-LLP2A SUV_max_ of iliac bones in healthy and MM participants at average of 240 min after injection of radiotracer. Mean and SD for SUV_max_ were 12.05 ± 2.0 for healthy participants (*n* = 5) and 25.62 ± 9.38 for MM patients (*n* = 3) (**P < 0.03, 2-tailed).

**FIGURE 4.**
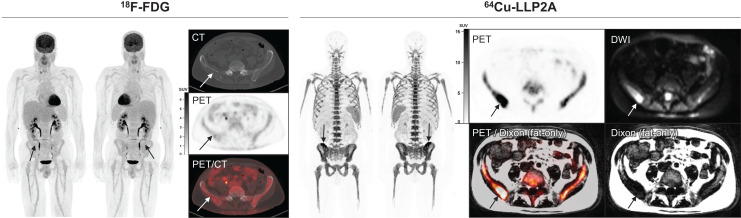
MM patient underwent PET imaging with ^64^Cu-LLP2A and ^18^F-FDG. On PET/CT, osteolytic lesion in right iliac bone (arrows) of MM patient had ^18^F-FDG uptake similar to background marrow. On PET/MRI, this same lesion (arrows) had ^64^Cu-LLP2A uptake above background marrow, corresponding to fat-replacing lesion on fat-only Dixon images and hyperintense lesion on DWI. In this lesion, ^64^Cu-LLP2A SUV_max_ was 29.5 with SUL_peak_ (per PERCIST) of 18.7; in comparison, ^18^F-FDG SUV_max_ was 2.9 with SUL_peak_ (per PERCIST) of 2.1. DWI = diffusion-weighted imaging.

Qualitative analysis showed that the best-quality images were obtained between 1 and 5 h after injection of the radiotracer. The images that were collected the next day, typically at around 24 h, were of relatively lower counts and higher image noise (Fig. 2). At an average of 240 min, the mean ± SD for the SUV_max_ of iliac bones in healthy volunteers (*n* = 5) was 12.05 ± 2.0, whereas it was 25.62 ± 9.38 for myeloma patients (*n* = 3) (2-tailed; *P* < 0.03) (Fig. 3). The BM SUV_max_ was measured as the average of lumbar vertebra 3–5 for most patients (Supplemental Fig. 5). Two participants had a new diagnosis of MM; both had negative findings on ^18^F-FDG PET/CT for diffuse or focal areas of increased uptake. However, one of these participants had a lytic lesion in the right iliac bone that was not ^18^F-FDG–avid and demonstrated focally intense ^64^Cu-LLP2A uptake. The scan of this MM participant (MMDM02) demonstrated diffuse moderate T1 hypointensity of the marrow in the spine and pelvis, similar to intervertebral disks and skeletal muscle, indicating diffuse marrow infiltration (Fig. 4). The second MM patient (MMDM01), who underwent PET/MRI, had recurrent MM with an increase in immunoglobulin M while on a regimen of elotuzumab. This subject had negative results on ^18^F-FDG PET/CT and on a bone survey. However, the MRI scan for MMDM01 showed heterogeneous mild T1 hypointensity of marrow in the spine and pelvis but not as T1 hypointense as in the intervertebral disks and skeletal muscle, a finding that might be attributable to red marrow and not sufficient to be called diffuse marrow infiltration on MRI. ^64^Cu-LLP2A-PET demonstrated an overall diffuse, moderately increased uptake throughout the BM in all of these 3 myeloma participants.

### Safety Evaluation

The mean and SD of the administered mass of ^64^Cu-LLP2A was 9.52 ± 1.33 μg (range, 6.9–11.7 μg). There were no adverse or clinically detectable pharmacologic effects in any of the participants. No changes in vital signs or in the results of laboratory studies or electrocardiography were observed. A comprehensive list of safety evaluation parameters and results is summarized in Supplemental Figure 6.

### Serum and Plasma Stability Study

Stability of ^64^Cu-LLP2A in blood samples was determined. Radioactive fragments (metabolites) were evaluated in the serum and plasma samples obtained from participants after they were injected with the radiotracer. Analytic radio–high-performance liquid chromatography was used to collect fractions, which were measured (radioactive counts) using the γ-counter. Data were normalized before plotting and analysis. Our original intent was to perform metabolite analysis from 0 to 4 h; however, the assay sensitivity was determined to be best for the time point at 1 h after injection. The low radioactivity counts after 1 h are likely due to the tracer’s small size and pharmacokinetics (rapid blood clearance). The rapid blood clearance diluted the signal to close to the baseline noise beyond 1 h. For patient convenience, we made the decision to limit the metabolite blood sampling to 1 h and amended the protocol accordingly. We previously performed ex vivo stability testing on human serum and demonstrated radiopharmaceutical stability up to 24 h. Here, the data showed that more than 90% of ^64^Cu-LLP2A was stable at 1 h, with the measured retention time of 5.5–7 min. The remaining radioactivity was accounted for by free ^64^Cu (elution time, 2–3 min) (Supplemental Fig. 7).

### Flow Cytometry Study

**FIGURE 5.**
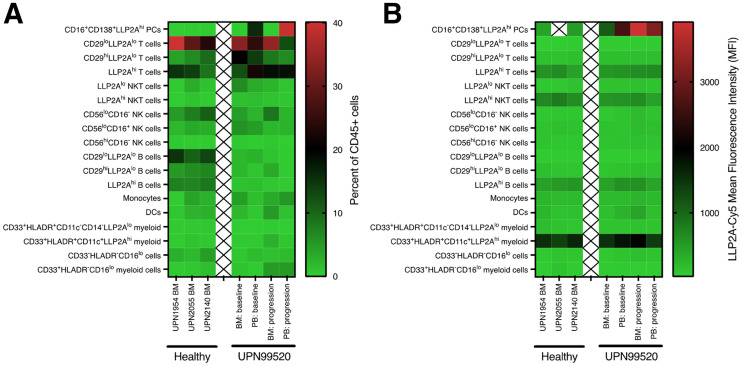
Flow cytometry analysis of LLP2A-Cy5 staining of human BM and peripheral blood cell subsets. (A) Heat map showing percentage of different cell subsets found within BM or peripheral blood (PB) of healthy participants from MM tissue bank (UPN1954, UPN2055, and UPN2140) or one of the MM patients (UPN99520/MMDM02) at baseline and after disease progression 4 mo later. (B) Heat map showing mean fluorescence intensity of LLP2A-Cy5 staining of different cell subsets.

The expression of activated VLA4 on hematopoietic cell populations within the BM or peripheral blood of 3 healthy participants from MM tissue bank (UPN1954, UPN2055, and UPN2140) and a patient with MM (MMDM02) (baseline and after disease progression 4 mo later) was examined by flow cytometry using LLP2A-Cy5 (19). Using a 14-color flow cytometry panel, we identified 18 different hematopoietic cell populations within these 7 samples (Supplemental Fig. 8). The BM mononuclear cells exhibited similar cellular distributions with the exception of fewer mature B cells in the patient with MM than in the healthy participants (Fig. 5A; Supplemental Fig. 9). In contrast, the peripheral blood samples from the patient with MM were enriched for CD138-positive plasma cells expressing high levels of CD16 and activated VLA-4, as measured with LLP2A-Cy5 (Fig. 5B; Supplemental Fig. 10). As we previously described, subsets of B cells, T cells, natural killer T cells, and myeloid cells expressing activated VLA-4 (LLP2A^hi^) were identified in both the healthy control and the MM samples (19).

## DISCUSSION

The landscape of MM pathogenesis and progression varies from patient to patient. Tumor heterogeneity, development of resistance to drugs, relapse of refractory disease, persistence of minimal residual disease, and variability in response are some of the hallmarks of MM. Molecular imaging has the ability to address some of the critical issues in management of MM patients by providing an accurate assessment of disease burden spatially, unambiguous staging, and quantitative and qualitative assessment of sites of disease, as well as detection of residual disease after treatment (27). Some outstanding challenges remain in the myeloma diagnostic tool kit: tissue biopsies are prone to sampling errors, serum assays can be confounding in cases of nonsecretory and heavily treated myelomas, conventional radiologic modalities are not sensitive for detecting osteolytic lesions, and molecular imaging using ^18^F-FDG has inherent limitations in MM. In recent years, different groups have assessed the utility of myeloma-specific agents targeted to myeloma proteins such as CD38 and CXCR4. Metabolic tracers such as ^18^F-FACBC (28) and ^11^C-acetate (29) have also been explored as alternatives to ^18^F-FDG. Although promising, the short 20-min radioactive half-life of ^11^C makes it a challenging radionuclide for routine use. The 12.7-h radioactive half-life of the positron emitter ^64^Cu, on the other hand, is a more viable option for wide clinical use.

Over 80% of myeloma patients present with skeleton-related events, and a significant number also experience pathologic fractures (30). Therefore, focusing on markers involved in the adherence, survival, and progression of myeloma cells in the BM is highly relevant. MM cells interact with the VCAM-1 expressed on the BM stromal cells and soluble fibronectin via VLA4 (31). Several studies have independently shown that VLA4 is overexpressed in MM cells relative to other cells and is an established marker of cell adhesion–mediated drug resistance.

Here, we describe, for the first time to our knowledge, the production of a VLA4-targeted clinical-grade tracer, ^64^Cu-LLP2A, under good-manufacturing-practice conditions in a cyclotron facility for use in human participants. The results of toxicity studies on rodents (no observed adverse effect level), and subsequently in patients, demonstrate the safety of injecting up to 15 μg of this tracer into patients. This level is not a limiting dose, but a reasonable starting point based on robust rodent imaging, toxicity, dosimetry, and safety data. The prerelease acceptance criteria, which included factors such as the pH, more than 90% radiochemical purity, more than 99% radionuclide purity, and an acceptable endotoxin result (<175 endotoxin units per total batch volume) was met for each imaging study. The postrelease quality control cell data also met the acceptance criteria for each study participant. These data validated the robustness of the tracer production and retention of bioactivity after the radiolabeling procedure.

Six healthy participants and 3 participants with a confirmed diagnosis of MM were injected with ^64^Cu-LLP2A. Metabolite analysis in these patients demonstrated that the tracer was more than 90% stable up to 1 h, with free copper identified as the only remaining metabolite. Accurate stability analysis beyond 1 h was not feasible because of sensitivity limitations. The calculated effective dose of ^64^Cu-LLP2A (0.036 mSv/MBq) is within the range of other reported copper radiopharmaceuticals (e.g., ^64^Cu-SARTATE [0.0204 mSv/MBq] (32), ^64^Cu-ATSM [0.036 mSv/MBq] (33), ^64^Cu-DOTA-AE105 [0.0284 mSv/MBq] (34), and ^64^Cu-DOTATATE [0.0315 mSv/MBq]) (35). The organ with the highest dose is the spleen, at a sex-averaged value of 0.142 mSv/MBq, followed by the red marrow (0.104 mSv/MBq) and bladder wall (0.094 mSv/MBq). As a comparison, the effective dose for ^18^F-FDG, the most widely used radiopharmaceutical for oncologic imaging, is 0.019 mSv/MBq (25).

The tracer pharmacokinetics in humans closely followed the rodent data, with rapid washout from blood and clearance via kidneys and bladder. As expected, there was relatively high uptake in the BM. The residence time in the BM was generally higher in women than in men and, overall, was higher in MM participants than in healthy participants. The SUV analysis of the BM in the iliac bones showed significantly higher values in the MM participants than in the healthy participants. The SD of SUVs was also higher in the MM participants across all time points. This finding points to the inhomogeneity and patchiness of the malignant BM in the MM patients as compared with healthy individuals (36). Comparison of image quality from early and late time points supports the selection of early time points—that is, 4–5 h after injection of the radiotracer—as optimal. This timing is advantageous for the convenience of myeloma patients, as they prefer same-day imaging because of the morbidity associated with the myeloma disease burden.

Takahashi et al. have demonstrated a quantitative metabolic parameter for ^18^F-FDG PET for assessing the intensity of bone involvement in MM (37). Li et al. proposed ^18^F-FDG uptake higher than liver as the positivity cutoff to discriminate between physiologic and pathologic uptake in the BM and defined 4 BM ^18^F-FDG uptake patterns (normal, focal, diffuse, and mixed) as reliable prognostic predictors of MM (38). In our study, we focused on the iliac bone SUV_max_ and SUV_mean_ and on SUVs normalized to liver and spleen. SUVs from the spine BM showed borderline statistical significance between MM and healthy participants (higher SUVs in MM participants). Further studies on a larger cohort are required to propose robust qualitative and quantitative metrics for ^64^Cu-LLP2A-PET. Other key variables to consider are the disease stage (precursor, newly diagnosed, relapsed, refractory, remission, or residual), genetic fingerprint, spatial distribution, BM inhomogeneity, age, and sex, as all these factors are known to impact myeloma pathogenesis (39).

## CONCLUSION

In this trial, we demonstrated that ^64^Cu-LLP2A can readily be synthesized with high quality and specific activity, is safe in humans, and has an acceptable radiation dosimetry, on a par with other ^64^Cu-labeled imaging agents and about twice higher than ^18^F-FDG, mainly because of the longer half-life of ^64^Cu. We demonstrated that phenotyping of the imaging signal using flow cytometry is complex yet feasible. Among the various discerning imaging features, the imaging data showed that there was uptake of ^64^Cu-LLP2A in the BM of healthy participants, as expected. To address this aspect of background uptake, we are working toward optimizing the imaging parameters to enhance the signal-to-background ratio in the malignant BM. The optimization approaches involve modulation of the imaging dose and molar activity as well as use of time-of-flight scanners for precise and sensitive imaging. Furthermore, in vitro and ex vivo studies evaluating the mechanisms of uptake, activation, retention, and efflux will be used to reduce background uptake. In our future ^64^Cu-LLP2A-PET imaging trials, we plan to incorporate a systems biology approach to integrate longitudinal clinical data with the imaging results.

## DISCLOSURE

This study was supported in part by U54CA199092, R42CA257797, R01 CA248493, R35 CA210084, R50CA211466, and R01CA176221. We thank the Mallinckrodt Institute of Radiology for pilot funds, the Foundation for Barnes–Jewish Hospital, and the Response Assessment Shared Resource Cores of the Alvin J. Siteman Cancer Center, supported in part by P30 CA91842. Monica Shokeen and Samuel Achilefu are cofounders of Sarya, LLC. No other potential conflict of interest relevant to this article was reported.

## Notes

KEY POINTS **QUESTION:** Is ^64^Cu-LLP2A-PET safe for use in humans? **PERTINENT FINDINGS:** This first-in-humans imaging trial demonstrated that ^64^Cu-LLP2A-PET is safe for use in MM patients. Preliminary findings support early imaging time points, which are amenable to patient comfort. **IMPLICATIONS FOR PATIENT CARE:** ^64^Cu-LLP2A-PET can inform on the unique biology of VLA4-positive malignant cells in MM patients, thus having potential to enhance diagnosis and guide therapy.
